# Negatively charged residues of the segment linking the enzyme and cytolysin moieties restrict the membrane-permeabilizing capacity of adenylate cyclase toxin

**DOI:** 10.1038/srep29137

**Published:** 2016-09-01

**Authors:** Jiri Masin, Adriana Osickova, Anna Sukova, Radovan Fiser, Petr Halada, Ladislav Bumba, Irena Linhartova, Radim Osicka, Peter Sebo

**Affiliations:** 1 Institute of Microbiology of the CAS, v. v. i., Prague, Czech Republic; 2 Faculty of Science, Charles University, Prague, Czech Republic

## Abstract

The whooping cough agent, *Bordetella pertussis*, secretes an adenylate cyclase toxin-hemolysin (CyaA) that plays a crucial role in host respiratory tract colonization. CyaA targets CR3-expressing cells and disrupts their bactericidal functions by delivering into their cytosol an adenylate cyclase enzyme that converts intracellular ATP to cAMP. In parallel, the hydrophobic domain of CyaA forms cation-selective pores that permeabilize cell membrane. The invasive AC and pore-forming domains of CyaA are linked by a segment that is unique in the RTX cytolysin family. We used mass spectrometry and circular dichroism to show that the linker segment forms α-helical structures that penetrate into lipid bilayer. Replacement of the positively charged arginine residues, proposed to be involved in target membrane destabilization by the linker segment, reduced the capacity of the toxin to translocate the AC domain across cell membrane. Substitutions of negatively charged residues then revealed that two clusters of negative charges within the linker segment control the size and the propensity of CyaA pore formation, thereby restricting the cell-permeabilizing capacity of CyaA. The ‘AC to Hly-linking segment’ thus appears to account for the smaller size and modest cell-permeabilizing capacity of CyaA pores, as compared to typical RTX hemolysins.

*Bordetella pertussis*, the etiological agent of the highly contagious respiratory disease known as pertussis, or whooping cough, secretes an adenylate cyclase toxin-hemolysin (CyaA, ACT, or AC-Hly). This belongs to the broad and important RTX (Repeats in ToXin) family of T1SS-secreted toxins of Gram-negative pathogens^1^. CyaA primarily targets host myeloid cells expressing complement receptor 3 (CR3), also known as the integrin α_M_β_2_, CD11b/CD18, or Mac-1^2^,^3^,^4^. Besides CR3-expressing cells, the toxin can also bind and detectably elevate concentrations of intracellular cAMP in a broad variety of other cells, including mammalian erythrocytes, on which CyaA exerts a hemolytic activity^5^,^6^. CyaA is a 1,706-residue-long (177-kDa) bifunctional protein that consists of an amino-terminal adenylate cyclase (AC) domain of about 400 residues and of an RTX cytolysin moiety (Hly) of about 1,300 residues^7^. The Hly moiety inserts into cellular membranes and mediates translocation of the enzymatic AC domain into cell cytosol, where the AC binds calmodulin and catalyzes conversion of ATP to cAMP, thereby subverting cellular signaling^3^,^8^,^9^,^10^ The AC appears to be a passive passenger and can be replaced by synthetic polypeptides that can also be delivered into cells by the Hly moiety^11^. In parallel, the Hly can form small cation-selective membrane pores that mediate efflux of potassium ions from cells^12^,^13^,^14^,^15^ and can cause colloid-osmotic cell lysis, such as hemolysis of erythrocytes^9^. The capacities of CyaA to penetrate cellular membranes, to form pores, and to deliver the AC domain into the cytosol of target cells, respectively, all depend on the covalent posttranslational fatty acylation of pro-CyaA at the ε-amino groups of the internal lysine residues Lys^983^ and Lys^860^, which modification is accomplished by a co-expressed protein toxin acyltransferase, CyaC^16^,^17^,^18^,^19^,^20^. Toxin activities further require loading of calcium ions into the numerous binding sites formed by the glycine- and aspartate-rich nonapeptide repeats of the RTX domain^21^,^22^,^23^.

Accumulated evidence suggests that at least two alternative and distinct conformers of CyaA co-exist and operate independently within the target cell membrane^24^,^25^,^26^,^27^. These CyaA conformers would exert two parallel and divergent activities, one accounting for translocation of the AC domain across cellular membrane, the other yielding formation of oligomeric cation-selective membrane pores. Moreover, AC domain translocation across lipid bilayer of cells does not require endocytosis of the toxin^5^ and proceeds directly across the cytoplasmic membrane with a very short half-time of about 30 seconds^6^. The process of AC domain translocation then appears to be driven by the negative potential on cellular membrane^28^,^29^ and proceeds in parallel to and independently of the formation of CyaA pores^25^. The exact path of translocation of the ~40 kDa AC domain polypeptide across the membrane bilayer and the structure of the CyaA translocon within the membrane remain, however, poorly defined.

The 3D structure of the AC domain in complex with calmodulin was solved^30^ and the structure-function relationships of the other domains of the RTX moiety are currently under intense exploration^17^,^20^,^23^,^24^,^26^,^31^,^32^,^33^,^34^,^35^,^36^,^37^,^38^,^39^. However, little is known about the structure and function of the ~100 residue-long segment that is located between residues 400 to 500 of CyaA and links its AC domain to the pore-forming domain of the Hly moiety. This CyaA segment has no homologs in the other toxins of the RTX family. It was previously proposed to be involved in translocation of the AC domain across the membrane, as AC domain translocation was ablated upon deletion of residues 375 to 485 of CyaA^40^,^41^. The ‘AC to Hly-linking segment’ then also appears to be involved in modulation of CyaA pore formation, as the truncated CyaA_ΔN489_ construct lacking the N-terminal residues 6–489 exhibits a strongly enhanced pore-forming and cell-permeabilizing activity^27^,^42^.

In the present work, we analyzed the structure and function of the ‘AC to Hly-linking segment’ and show that its clusters of negatively charged residues modulate the pore-forming activity of CyaA, whereas its positively charged arginine residues may be involved in enabling AC domain translocation across target cell membrane.

## Results

### The ‘AC to Hly-linking segment’ consists of α-helices that insert into lipid bilayer

Recently a synthetic peptide comprising residues 454 to 484 of CyaA was found to bind and permeabilize liposomal membranes^41^. We therefore examined the membrane-penetrating capacity of the entire linker segment that connects the invasive AC enzyme to the hydrophobic pore-forming domain of CyaA. Towards this aim, the polypeptide consisting of residues 411 to 490 of CyaA was fused to the TEV protease recognition sequence at the C-terminal end of a glutathione S-transferase moiety, fused N-terminally to a double 6xHis affinity tag. The resulting d6His-GST-CyaA_411–490_ protein was purified close to homogeneity by immobilized metal affinity chromatography on Ni-NTA agarose (Supplementary Fig. S1) and its membrane-inserting capacity was assessed by incubation with unilamellar liposomes. To identify the potential liposome membrane penetrating segments, the unbound d6His-GST-CyaA_411–490_ molecules were removed by washing and the protein molecules bound to liposomes were digested by excess of trypsin. Peptides not inserted into the liposomal membrane were removed by three successive washes of the liposomes in buffer, in 0.1 M Na_2_CO_3_ (pH 10.5) and in water, respectively. The liposome-inserted peptides were then extracted into 0.2% TFA and identified by MALDI FT-ICR mass spectrometry. As documented in Table 1, peptides that were not tightly associated with the lipid bilayer membrane were efficiently stripped-off by washing of the liposomes with 0.1 M Na_2_CO_3_ (pH 10.5), as most of the GST-derived tryptic peptides were undetectable in the extract. In contrast, peptides covering the entire sequence of the CyaA_411–490_ linker segment were unambiguously identified in the extract together with four tryptic peptides derived from the C-terminus of GST (c.f. Table 1, Supplementary Table 1). Hence, the entire linker segment (CyaA residues 411 to 490) interacted tightly with the liposomal membrane and entrained into the lipid bilayer also the C-terminal portion of GST.

**Figure 1:**
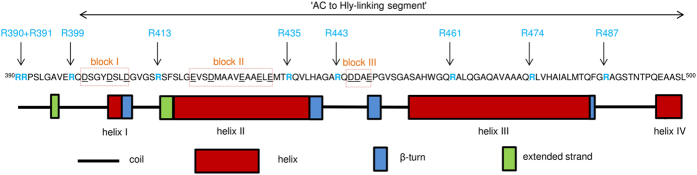
Schematic representation of predicted secondary structures in the in the ‘AC to Hly-linking segment’ that connects the invasive AC and pore-forming domains of CyaA. Prediction was performed using the SOPMA software^43^. Three blocks of negatively charged residues are highlighted by rectangles and negatively charged residues are underlined. Positions of mutagenized arginine residues are represented by arrows.

**Figure 2:**
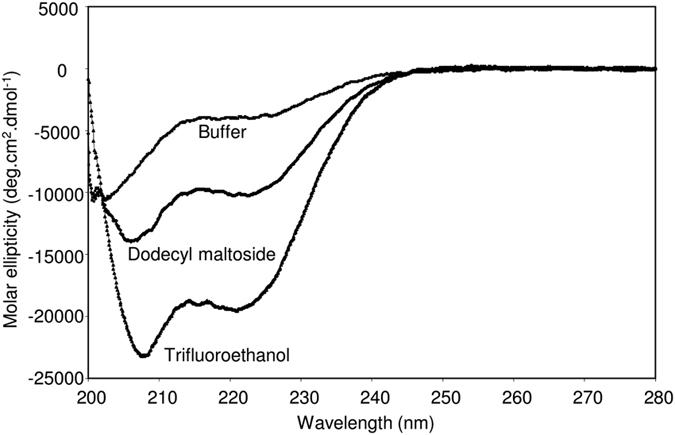
The CyaA_411–490_ segment adopts an α-helical structure in the presence of dodecyl-maltoside and trifluoroethanol. The CyaA_411–490_ segment was rapidly diluted with 5 mM Tris-HCl (pH 7.4), 5 mM Tris-HCl (pH 7.4), 1% n-dodecyl-β-D-maltoside, or 5 mM Tris-HCl (pH 7.4), 50% trifluoroethanol to a final protein concentration of 100 μg/ml. The samples were measured for wavelengths from 200 to 280 nm at 20 °C.

Secondary structure prediction analysis of the segment by the SOPMA algorithm^43^ indicated that two long *α*-helices may form between residues 416 to 438 and 455 to 484 of CyaA, respectively (Fig. 1). To test the prediction, the CyaA_411–490_ fragment was separated from the d6His-GST moiety by TEV protease digestion and purified close to homogeneity under denaturing conditions in 8 M urea using Ni-NTA agarose that retained the d6His-GST fragment (Supplementary Fig. S1). As assessed by CD spectroscopy in Fig. 2, the purified CyaA_411–490_ polypeptide remained unstructured upon dilution in 5 mM Tris-HCl (pH 7.4) buffer, exhibiting in the aqueous environment a negative band at 200 nm. Upon addition of agents mimicking membrane environment, such as 50% trifluoroethanol, or of the micelles of the non-ionic detergent dodecyl maltoside (1%), a strong increase of the intensity of bands at 222 and 208 nm (Fig. 2) was observed, indicating formation of *α*-helical secondary structures in the CyaA_411–490_ segment (Supplementary Table 2).

### Positively charged arginine residues within the ‘AC to Hly-linking segment’ are involved in AC domain translocation across the target cell membrane

**Figure 3:**
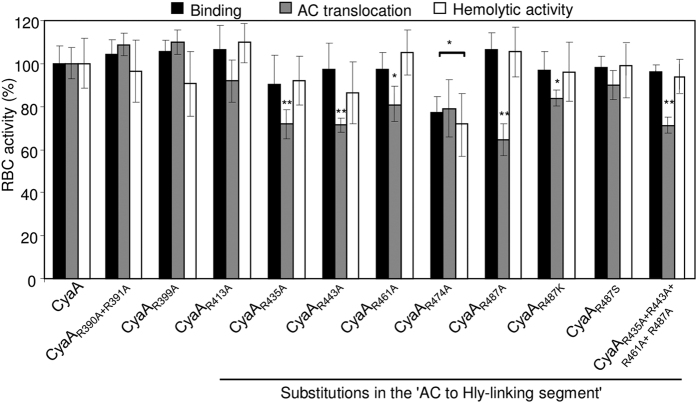
Substitutions of the positively charged arginine residues in the ‘AC to Hly-linking segment’ decrease the invasive capacity of CyaA. Sheep erythrocytes (5 × 10^8^/ml) were incubated at 37 °C with 1 μg/ml of the purified CyaA proteins and after 30 min, aliquots were taken for determinations of the cell-associated AC activity and of the AC activity internalized into erythrocytes and protected against digestion by externally added trypsin. The hemolysis was measured at 541 nm as hemoglobin release after 220 min of toxin incubation (10 μg/ml) with erythrocytes. All activities are expressed as percentages of intact CyaA activity taken as 100% and represent the average values ± standard deviations from at least three independent determinations, performed in duplicate with two different toxin preparations (n = 6–10). Asterisks indicate values significantly different from intact CyaA (*p < 0.05; **p < 0.001).

The above results indicated that the segment between residues 400 and 500 of CyaA contains membrane-interacting α-helices. Inspection of the primary sequence of the segment revealed presence of three clusters of negatively charged aspartate and glutamate residues in its N-terminal half (Fig. 1, blocks I to III). No lysine residues and only six positively charged arginine residues are then contained within this segment. Of them, two are located in its C-terminal half (R461 and R474) and were previously invoked as being involved in the membrane-destabilizing activity of the peptide consisting of residues 454 to 484 of CyaA^41^. We thus tested whether these arginine residues play a role in insertion of the toxin into and translocation of its AC domain across the target cell membrane. The six arginine residues of the ‘AC to Hly-linking segment’ were substituted by alanine residues (e.g. R413A, R435A, R443A, R461A, R474A and R487A). As a control, the N-terminally clustered arginine residues at positions 390, 391 and 399 were substituted (e.g. R390A + R391A, R399A). The CyaA variants were produced in *E. coli,* purified close to homogeneity and characterized for the capacity to bind, penetrate and lyze sheep erythrocytes. As shown in Fig. 3, the CyaA toxins with individual Arg > Ala substitutions at positions 413, 435, 443, 461 and 487, respectively, exhibited full capacity to bind and permeabilize erythrocytes (hemolytic activity), with their specific activities differing by less than 10% from the activities of intact CyaA. However, with the exception of the CyaA_R413A_ construct, the specific capacity of these mutant toxins to translocate their AC domains across the erythrocyte membrane was selectively reduced by about 20% to 40% compared to that of intact CyaA. Moreover, the alanine substitution of the arginine residue at position 474 (CyaA_R474A_) significantly reduced specific toxin-binding, AC domain-translocating and hemolytic activities of CyaA_R474A_, respectively. In contrast, the substitutions of the arginine residues clustered at the N-terminal end of the linking segment (R390A + R391A, R399A) had no discernible impact on cell-binding and cell-invasive activities of the toxin (Fig. 3). It appears, hence, that the arginine residue at position 474 is specifically involved in interaction of CyaA with the membrane of target cells. This would go well with its central location within a hydrophobic α-helical structure shown to destabilize lipid bilayer membranes^41^. Interestingly, substitution of the adjacent arginine at position 487 by a charge-preserving lysine residue (CyaA_R487K_), or by a hydrophilic serine residue (CyaA_R487S_), had a lower impact on the ability of the toxin to translocate the AC domain across lipid bilayer than had the alanine substitution of the same residue in the CyaA_R487A_ construct. Surprisingly then, as shown in Fig. 3, no additive effect of combining of four Arg > Ala substitutions in the CyaA_R435A+R443A+R461A+R487A_ construct was observed. The specific cell-binding, cell-invasive and hemolytic activities of the construct with multiple substitutions were comparable to the activities of toxins bearing single substitutions. This suggests that the positively charged arginine residues R435, R443, R461 and R487 do not synergize and may play redundant roles in the course of CyaA interaction with target cells and AC domain translocation across their membrane.

### Negatively charged residues located in the ‘AC to Hly-linking segment’ modulate formation of CyaA pores

**Figure 4:**
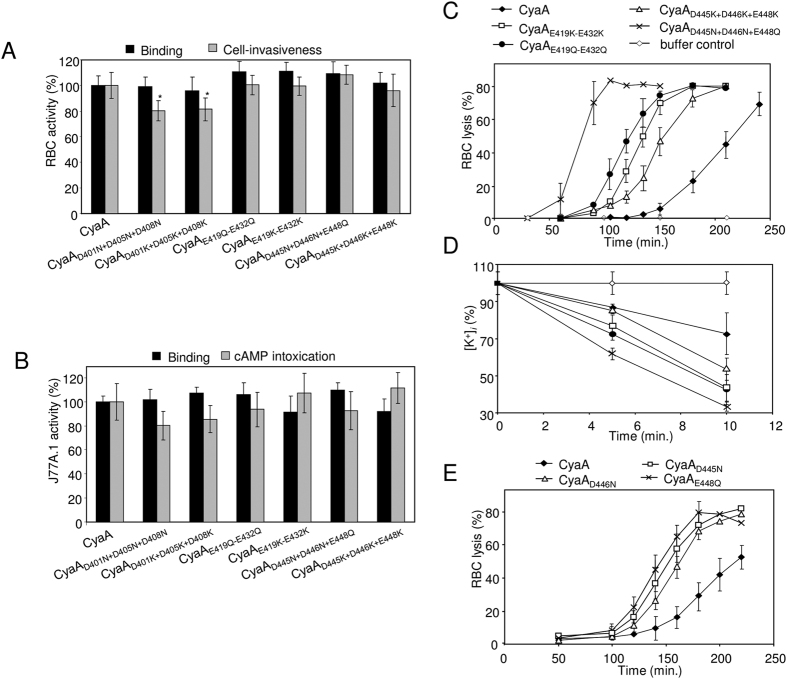
Substitutions of glutamate and aspartate residues in blocks II and III of the ‘AC to Hly-linking segment’ enhance pore-forming capacity of CyaA without altering AC translocation. (**A**) Sheep erythrocytes (5 × 10^8^/ml) were incubated at 37 °C with 1 μg/ml of the purified CyaA proteins for 30 min. Aliquots were taken for determination of the cell-associated AC activity and of the AC activity internalized into erythrocytes and protected against digestion by externally added trypsin, respectively. The activities are expressed as percentages of intact CyaA activity (100%) and represent average values ± standard deviations from at least three independent determinations performed in duplicate with two different toxin preparations (n ≥ 6). Asterisks indicates values significantly different from intact CyaA (*p < 0.05). (**B**) Binding of the CyaA proteins to J774A.1 cells (10^6^) was determined as the amount of total cell-associated AC enzyme activity upon incubation of cells with 1 μg/ml of the protein for 30 min at 4 °C. cAMP intoxication was assessed by determining the intracellular concentration of cAMP generated in cells upon incubation of J774A.1 cells (2 × 10^5^) with four different toxin concentrations from within the linear range of the dose-response curve (100, 50, 25, 10 ng/ml). The percentage of cAMP accumulation in cells at each toxin concentration was calculated, taking cAMP values for intact CyaA as 100%. (**C,E**) Sheep erythrocytes (5 × 10^8^/ml) in TNC buffer were incubated at 37 °C in the presence of intact CyaA or of its mutant variants (10 μg/ml) or the urea buffer control. Hemolytic activity was measured as the amount of released hemoglobin by photometric determination (A_541_ _nm_). Error bars represent standard deviations from four independent measurements performed with two different toxin preparations. (**D**) J774A.1 macrophages (10^6^/ml) in modified HBSS buffer without KCl were incubated with 500 ng/ml of intact CyaA or of its mutant variants, or the urea buffer only. The reaction was stopped at 5 or 10 min of incubation, the cells were pelleted by centrifugation, washed in modified HBSS and lysed by deionized water. Lysates were diluted with deionized water and analyzed by ICP–MS. Error bars represent standard deviations of four samples measured in two independent experiments.

Previously, negatively charged residues within the predicted transmembrane α-helices of the adjacent hydrophobic domain (residues 500 to 700) were shown to play a crucial role in translocation of the AC domain across the membrane, as well as in the formation and ion selectiveness of CyaA pores^24^,^26^. We thus examined whether the negatively charged residues of the AC-Hly linking segment play a role in CyaA toxin activities. Towards this aim, the aspartate and glutamate residues of the three clusters (block I: D401 + D405 + D408; block II: E419 + D422 + E427 + E430 + E432 (=E419-E432); and block III: D445 + D446 + E448; Fig. 1) were replaced by neutral asparagine or glutamine residues, or by oppositely charged lysine residues, respectively. The toxin variants were purified close to homogeneity and their specific hemolytic and cytotoxic activities were assessed. Two cell types were used for this purpose, where sheep erythrocytes served as model cells devoid of the toxin receptor CR3 and the mouse J774A.1 macrophage cells, expressing high amounts of CR3, served as model phagocyte targets of the toxin. As documented in Fig. 4A,B, the charge neutralizing (D/E > N/Q) or reversing (D/N > K) substitutions of aspartate and glutamate residues within blocks II and III had essentially no impact on the cell-binding or cell-invasive activities of the toxin on erythrocytes or J774A.1 cells. The CyaA_D401N+D405N+D408N_ or CyaA_D401K+D405K+D408K_ toxin variants, bearing substitutions in block I, exhibited only a moderately enhanced specific cell-permeabilizing (hemolytic) activity (Supplementary Fig. S2) and their capacity to translocate the AC domain across the membrane of erythrocytes, or J774A.1 cells, was only slightly (~20%) reduced (Fig. 4A,B). In contrast, the substitutions in the block II and III strongly enhanced the specific hemolytic activity of the respective toxins on erythrocytes, with the combination of neutral D445N + D446N + E448Q substitutions in block III exhibiting the highest impact (Fig. 4C). In agreement with their enhanced hemolytic potencies, all the CyaA mutants with substitutions in blocks II and III exhibited also a strongly enhanced capacity to permeabilize the CR3-expressing J774A.1 macrophage cells, provoking a time-dependent drop of intracellular [K^+^]_i_ concentration in J774A.1 cells, as shown in Fig. 4D. Moreover, multiple substitutions of the aspartate and glutamate residues of block III (D445 + D446 + E448) by neutral and hydrophilic serine residues had no impact on the cell-binding or cell-invasive capacities of the CyaA_D445S+D446S+E448S_ toxin (Supplementary Fig. S4A). Further, the serine substitutions enhanced the specific hemolytic activity of the mutated toxin (Supplementary Fig. S4B). The truly hyperhemolytic phenotype of the CyaA_D445N+D446N+E448Q_ toxin then resulted from a synergic effect of the three substitutions, as the toxins with individual D445N, D446N and E448Q substitutions exhibited only mildly enhanced specific hemolytic activities (Fig. 4E). The singly mutated toxins were also intact in their cell-binding and cell-invasive activities (Supplementary Fig. S3). These results, hence, suggest that the negatively charged residues of blocks II and III play a specific role in modulation of the pore-forming activity of CyaA, without being involved in the membrane penetrating and AC domain translocating activity of the toxin.

### Clusters of charged side chain groups in blocks II and III of the ‘AC to Hly-linking segment’ restrict the size of CyaA pores

**Figure 5:**
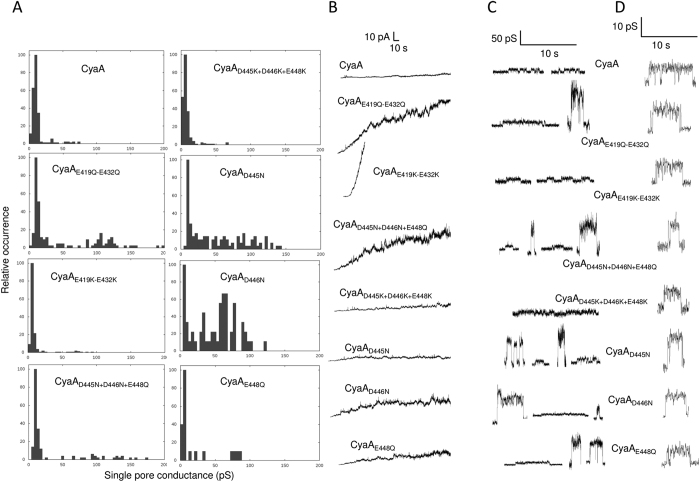
The conductance of single CyaA pores and membrane activity is affected by substitutions in blocks II and III. (**A**) Single-pore conductance of intact CyaA or its mutant variants (1 nM) was determined in 150 mM KCl, 10 mM Tris-HCl and 2 mM CaCl_2_ (pH 7.4) at 25 °C and membrane potential −50 mV. (**B**) Single-pore recordings of asolectin/n-decane membranes in the presence of 1 nM purified CyaA variants. The applied membrane potential was −50 mV and the temperature was 25 °C. (**C**) Representative recordings of the most frequent types of observed pores (the noise was suppressed by 50 Hz low-pass filter). (**D**) Detail of most frequent conductance states (filtered at 10 Hz). The presented events were acquired on several different asolectin membranes for each toxin. Measurement conditions: 150 mM KCl, 10 mM Tris, 2 mM CaCl_2_, pH 7.4, toxin concentration 0.1–1 nM, transmembrane potential −50 mV.

To ascertain the molecular basis of the enhanced cell-permeabilizing capacity of toxins with substitutions of negatively charged residues of block II and III, we next determined the frequencies of formation, the distribution of conductances and lifetimes of the pores formed by these constructs in black lipid bilayer membranes. As shown in Fig. 5A, in 150 mM KCl and 2 mM CaCl_2_ at pH 7.4 the single-pore units of intact CyaA in soybean asolectin membranes exhibited a conductance ranging from 8 to 15 pS, with the most frequent conductance being 10.4 ± 3.6 pS. The CyaA_E419K−E432K_ and CyaA_D445K+D446K+E448K_ mutants formed pores of similar conductance 9.5 ± 3.3 pS and 9.0 ± 3.3 pS (Fig. 5A) but with much enhanced propensity (Fig. 5B, Table 2). A larger dispersion of conductance unit sizes was observed for the CyaA_E419Q−E432Q_ and CyaA_D445N+D446N+E448Q_ variants bearing neutral residue substitutions. These constructs formed pores with enhanced frequency (Fig. 5B and Table 2), with the most frequent conductances being 10.2 ± 1.6 pS and 11.6 ± 2.7 pS, as for intact CyaA, but occasionally formed also pore units with conductances higher than 100 pS (*c.f.* Fig. 5A). A larger dispersion of conductance unit sizes was then observed also for the CyaA toxins carrying single substitutions of residues 445, 446 or 448 from block III (Fig. 5A), or a combined triple serine substitution (CyaA_D445S+D446S+E448S_, Supplementary Fig. S4C and S4D). The CyaA_D446N_ and CyaA_E448Q_ proteins then also formed pores with higher frequency than intact CyaA (Fig. 5B).

As documented in Table 2, mean lifetimes of pores formed by most of the constructs were similar to that of intact toxin pores and ranged between 1.2 to 1.8 seconds. The outliers were the CyaA_E419Q-E432Q_ mutant, which formed pores with a longer mean lifetime of ~2.4 second and the CyaA_D445N_ toxin, which formed pores with a shorter mean lifetime of ~0.5 second. None of the mutants, however, exhibited a propensity to form any substantially longer-living pores, such as those observed previously for CyaA bearing neutral or charge-reversing substitutions of the glutamates 516 and 581^12^,^24^,^26^. The substitutions of aspartate and glutamate residues of blocks II and III thus affected the propensity of formation and the distribution unit pore conductance (size) of formed CyaA pores, strongly enhancing the cell-permeabilizing capacity of the toxin.

### Enhancement of pore-forming capacity enhances cytotoxicity of CyaA

**Figure 6:**
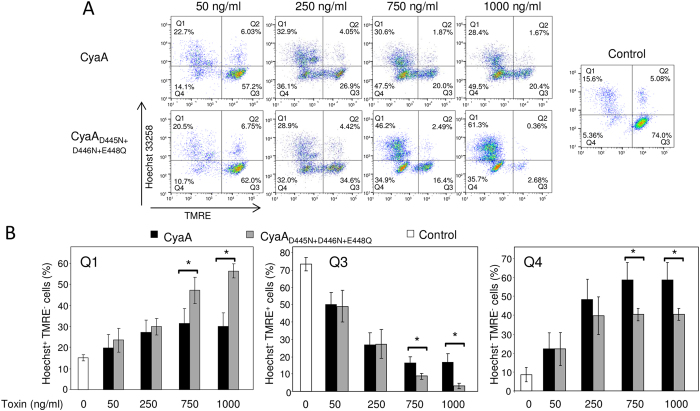
Enhanced pore-forming activity potentiates the cytotoxic action of fully invasive CyaA_D445N+D446N+E448Q_ mutant on macrophages. (**A**) J774A.1 cells (2 × 10^5^/well) labeled with TMRE (40 nM) were incubated for 2 hours with indicated concentrations of CyaA, CyaA_D445N+D446N+E448Q_ or buffer control. The J774A.1 cells were stained with Hoechst 33258 (0.5 μg/ml) and the live (Hoechst^−^ TMRE^+^) and necrotic (Hoechst^−^ TMRE^−^) cells were detected by FACS. (**B**) The results represent average values from four independent experiments. *, statistically significant differences (p < 0.05).

Toxic action of CyaA on phagocytes results from the synergy of cytotoxic signaling of toxin-produced cAMP with the cell-permeabilizing action of the CyaA pores^8^,^44^,^45^. Therefore, we examined the specific cytotoxic activity of the fully cell-invasive CyaA_D445N+D446N+E448Q_ mutant that exhibits a strongly enhanced hemolytic capacity. J774A.1 macrophage cells expressing the toxin receptor CR3 were loaded with the TMRE sensor of mitochondrial potential, the cells were exposed for 2 hours to a range of intact and “hyperhemolytic” CyaA_D445N+D446N+E448Q_ toxin concentrations and the proportions of live and necrotic cells were determined by flow cytometry. As shown in Fig 6, at toxin concentrations of 50 and 250 ng/ml, respectively, there was no significant difference in the cytotoxic potencies of the intact and “hyperhemolytic” CyaA_D445N+D446N+E448Q_ that yielded comparable proportions of live (quadrant Q3), necrotic (quadrant Q1) or non-permeabilized but mitochondrial membrane potential-depleted cells (quadrant Q4), respectively. When compared at 750 or 1000 ng/ml, however, the action of CyaA_D445N+D446N+E448Q_ yielded a significantly higher proportion of necrotic J774A.1 cells (Q1), with a corresponding reduction of numbers of cells remaining alive (Q3), or non-permeabilized but devoid of mitochondrial potential (Q4). Hence, at higher toxin concentrations the selective increase of specific cell-permeabilizing capacity of CyaA translated into an enhanced specific cytotoxic potency towards CR3-expressing cells.

## Discussion

**Figure 7:**
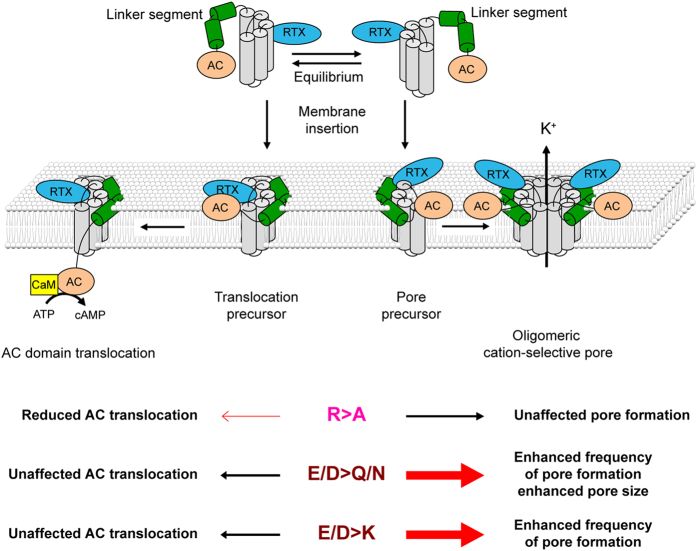
Schematic model of CyaA action on target membrane. CyaA penetrates the cytoplasmic membrane and would employ two distinct conformers to accomplish its multiple actions within the membrane. One conformer would form cation-selective pores that permeabilize the membrane bilayer for efflux of cytosolic potassium ions. The other conformer would translocate the AC enzyme domain into cells across the plasma membrane and catalyze conversion of cytosolic ATP to cAMP. As shown here, the ‘AC to Hly-linking segment’ can insert into the cell membrane and would adopt an α-helical conformation with the axis of the helices being parallel to the plane of the lipid bilayer^41^. The linker segment containing the positively charged arginine residues would exhibit membrane interacting properties and would induce a local destabilization of the lipid bilayer. This would subsequently facilitate translocation of the AC domain across the lipid bilayer^41^. As shown here, CyaA mutants carrying arginine to alanine substitutions (R > A) in the central and C-terminal part of the linker segment bind the target membrane and form membrane pores with equal efficacy as intact CyaA (except for CyaA_R474A_), while their ability to translocate the AC domain across the membrane is reduced. Replacement of the negatively charged residues of blocks II and III (E/D > Q/N, E/D > K) yields “hyperhemolytic” CyaA variants. Membrane-inserted pore precursors of the CyaA_E419Q−E432Q_ and CyaA_D445N+D446N+E448Q_ mutants assemble with high frequency, thereby giving rise to the highly increased pore-forming activity. Moreover, the pore precursors of CyaA_E419Q−E432Q_ and CyaA_D445N+D446N+E448Q_ constructs exhibit not only an increased propensity to form membrane pores but also form pores with higher conductance states. The ability of these mutants to translocate the AC domain across the membrane of target cells, however, is not affected. The model and the sizes of individual domains of CyaA are not drawn to scale. RTX, calcium binding RTX domain, AC, invasive AC domain, K^+^, potassium efflux, CaM, calmodulin.

CyaA and the MARTX proteins belong to the unique group of enzymatically active bacterial toxins capable to deliver their enzymatically active moieties into target cell cytosol directly across the cytoplasmic membrane of cells^5^,^46^. Our current model predicts that CyaA likely adopts at least two independent and parallel/competing conformations within target membrane (Fig. 7). Recently, soluble monomeric CyaA toxin could be isolated by size exclusion chromatography^47^ and the sum of available data suggests that one of CyaA conformers would account for formation of oligomeric CyaA pores that elicit potassium efflux from cells. The other CyaA conformer would then account for membrane insertion of a toxin translocation precursor and delivery of the AC domain into the cytosol of cells. The AC translocation process appears to depend on transient opening of a path for influx of extracellular Ca^2+^ions across cell membrane and can occur in the absence of potassium efflux and independently of formation of the cell-permeabilizing oligomeric toxin pores^14^,^24^,^26^,^27^,^48^,^49^. The balance between the action of the two conformers on cellular membrane can then be modulated by substitutions of glutamate residue pairs localized in the predicted transmembrane segments of the pore-forming domain of CyaA^24^,^25^,^26^. Here we show that the propensity of CyaA pore precursor assembly into oligomeric pores is controlled by the negative charges of aspartate and glutamate residues clustered in blocks II and III within the N-terminal half of the ‘AC to Hly-linking segment’. These charges appear to play a role in restriction of size of the CyaA pores and in control of the frequency of pore formation, but do not seem to play a role in AC domain translocation across cellular membrane. The negatively charged structure in the N-terminal half of the linking segment thus appears to be selectively involved in regulation of the pore-forming activity of CyaA, possibly by down-modulating the oligomerization propensity of CyaA through electrostatic repulsion of CyaA pore subunits. The positively charged residues in the C-terminal half of the ‘AC to Hly-linking segment’ then appear to be involved in facilitation of AC domain translocation across the membrane.

The exact underlying mechanism and the specific structures involved in translocation of the ~40 kDa AC enzyme moiety of CyaA across the lipid bilayer remain elusive. Translocation of the AC domain appears to depend on negative membrane potential^28^,^29^ and requires a net positive charge of the AC domain^50^. Studies on the use of CyaA as conveyor of antigens into cytosol of antigen presenting cells show that in order to be efficiently translocated across target cell membrane by the Hly moiety, the linked AC domain, or the antigen substituting it, need to bear an overall net positive charge^11^,^51^,^52^. This would suggest that the translocated polypeptide may be conducted along a negatively charged hydrophilic surface across the membrane lipid bilayer, which would possibly comprise the amphipathic transmembrane α-helices formed by residues 502 to 522 and 565 to 591 of CyaA^24^,^26^. Indeed, helix-breaking or charge-reversing substitutions in these segments (E509K, E516K and E581K) had a devastating impact on the capacity of CyaA to translocate the AC across the membrane and potentiated the pore-forming capacity of CyaA. In contrast, either the here-reported reduction of the net negative charge of the N-terminal half of the ‘AC to Hly-linking segment’ from −7 to −1 (e.g. in CyaA_D445K+D446K+E448K_), or the reversal of its overall charge to +3 in the CyaA_E419K-E432K_ construct, had essentially no impact on the capacity of CyaA to translocate its AC domain across erythrocyte or macrophage membranes. Hence, the clusters of negatively charged residues in the N-terminal half of the ‘AC to Hly-linking segment’ are clearly not part of the conducting path along which the AC domain translocates across the lipid bilayer.

Recently, a synthetic α-helical peptide consisting of the residues 454 to 485 of the C-terminal half of the ‘AC to Hly-linking segment’ was shown to penetrate into the outer leaflet of the lipid bilayer of liposomes^41^. The authors proposed that the segment may be inducing local membrane destabilization and could facilitate AC domain translocation across plasma membrane of target cells. This would go well with the here-reported identification of six membrane-inserted peptides of d6His-GST-CyaA_411–490_ bound to liposomal membrane (Table 1). These linker segment peptides were neither washed-out from the membrane of trypsin-digested liposomes with neutral buffers, nor could be extracted into 0.1 M sodium carbonate at pH 10.5, which is an established method for removal of peripherally attached membrane proteins^53^. The CyaA_411–490_ segment, hence, inserted to large extent into the lipid bilayer of liposomes. The accessibility of the arginine residues to trypsin then indicates that the linker segment was tightly associated with, or was inserted into the membrane in parallel to its plane, but was not entirely buried within the outer leaflet of the liposomal membrane. Four of the identified peptides (444–461, 462–474, 475–487 and 462–490) then encompass the previously studied membrane-interacting α-helical peptide_454–485_ of CyaA^41^. Interpretation of these data, however, still deserves some caution. We have previously observed that due to absence of the membrane potential critical for transmembrane insertion and correct positioning of CyaA segments, the liposomal membrane system does not reproduce the interactions of CyaA with cellular membrane truly enough^54^. Moreover, the extent and topology of membrane interactions of the isolated 80-residue-long linker segment and the membrane interactions made by the same structure in the context of the full-length toxin molecule, may differ.

The peptide 454 to 485 of CyaA does not exhibit features of cell penetrating peptides. Nevertheless, its two positively charged arginine residues (R461 and R474) were proposed to be involved in recruitment of anionic lipids that would be inducing non-lamellar lipid structures and would destabilize the lipid bilayer, thus facilitating AC domain translocation^41^. Our results identify four arginine residues localized within the ‘AC to Hly-linking segment’ (R435, R443, R461 and R487) as good candidates for involvement in such action. Alanine substitutions of these residues selectively reduced the capacity of CyaA to translocate its AC domain across membrane and did not affect the overall membrane-inserting and pore-forming activities of CyaA. Moreover, replacement of the arginine residue at position 487 by a positively charged lysine, or a hydrophilic serine residue, reduced the AC-translocating activity of CyaA notably less than a hydrophobic alanine substitution. The incomplete restoration of the AC-translocating activity by the conservative lysine substitution would plausibly be explained be the requirement for a larger guanidinium group of the arginine residue. This would form stronger electrostatic interactions and a higher number of hydrogen bonds with the phosphates of phospholipid headgroups than an amino group of a lysine residue side chain^55^,^56^. The strong and highly selective impact of the hydrophobic alanine substitution of arginine 487 would then indicate that the hydrophilic nature of this residue may play a structural role in toxin activity.

CyaA forms cation-selective membrane pores of an inner diameter of 0.6 to 0.8 nm and the same pore characteristics were also observed for pores formed by the CyaA_ΔAC_ variant that lacks the 373 N-terminal residues of the cell-invasive AC domain^12^. When most of the ‘AC to Hly-linking segment’ is removed by N-terminal truncation up to residue 489, the CyaA_ΔN489_ construct exhibits a high propensity to form larger pores than intact CyaA^27^,^42^. In the light of the here-presented data, it is plausible to speculate that the increased size and frequency of formation of CyaA_ΔN489_ pores may be due to a combination of effects resulting from removal of the negative charges comprised within the ‘AC to Hly-linking segment’ that modulate the pore-forming propensity of CyaA, as shown here. The truncation also may eliminate a structure encumbering the CyaA pore and restricting its inner diameter.

Compared to other RTX toxins, indeed, CyaA is a less potent hemolysin. It forms smaller pores than any other of the so far characterized RTX cytolysins, which are all devoid of homologues of the ‘AC to Hly-linking segment’ at the N-terminal ends of their pore-forming domains^9^,^57^. The best characterized RTX hemolysins, such as *E. coli* HlyA or *Actinobacillus pleuropneumoniae* ApxIA, form larger pores with estimated inner diameters of 1.5 to 2 nm and generate pore units with about an order of magnitude higher conductance than CyaA^12^,^42^. It is thus plausible to speculate that the membrane-interacting ‘AC to Hly-linking segment’, or its part, may act as a sort of a molecular iris on top of, or around the CyaA pore. The negative charges of glutamate and aspartate residues clustered between residues 419 to 448 might, by electrostatic interactions, dictate the positioning (immobilization) of the transmembrane segments that harbor glutamates 516 and 581 and control the size, frequency of formation, lifetime and ion selectiveness of CyaA pores^24^,^26^. Such electrostatic interaction between negatively charged residues of CyaA segments might then prevent formation of “looser” and potentially less ion-selective CyaA assemblies. Such larger pores/lesions of higher conductance appear, indeed, to be formed by the CyaA_E419Q-E432Q_, CyaA_D445N+D446N+E448Q_ or CyaA_D445S+D446S+E448S_ mutants bearing neutral substitutions that eliminate the negative charges of block II and III of the ‘AC to Hly-linking segment’. Similar effect is, indeed, observed upon deletion of the negatively charged segment in the CyaA_ΔN489_ construct^27^,^42^. Intriguingly, replacement of the negative charges in this segment by positive charges of lysine residues had essentially no impact on the size (conductance) of pores formed by the CyaA_E419K-E432K_ and CyaA_D445K+D446K+E448K_ constructs. The charge reversal only enhanced the frequency of formation of CyaA pores. This induces the hypothesis that distribution of charges and electrostatic interactions might control positioning of critical CyaA pore segments in the membrane. It appears plausible to speculate that immobilization and correct positioning of the critical pore segments, bearing the glutamate residues 516 and 581, might be equally well assured by electrostatic attraction, resulting from introduction of positively charged residues, as it results from electrostatic repulsion by the negative charges within blocks II and III of the ‘AC to Hly-linking segment’ of intact CyaA.

The reduced specific cell-permeabilizing activity of the RTX hemolysin moiety of CyaA may then represent an evolutionary fine-tuning of CyaA toxin activities for the sake of a potent immunosuppressive action in the course of host airway colonization by *Bordetellae*. In contrast to other RTX cytolysins, which incapacitate host leukocytes by forming larger pores in their membranes, the immunosuppressive action of CyaA relies primarily on the AC domain-catalyzed production of cAMP. This hijacks the cellular signaling mechanisms of CR3-expressing phagocytes and ablates the bactericidal activities of neutrophils and macrophages that infiltrate *Bordetella-*infected tissues^58^. In the second line, the cAMP-mediated downregulation of pro-inflammatory IL-12 and TNF-α production and the upregulation of anti-inflammatory IL-10 cytokine release by myeloid cells, would skew the adaptive immune responses to infection and protract bacterial colonization. In contrast, the pore-forming activity of CyaA was shown to elicit potassium efflux and thereby contributes activation of the p38 and JNK kinases in toxin-permeabilized monocyte-derived cells^59^. This then synergizes with TLR-mediated signaling of bacterial components towards promoting NALP3 inflammasome assembly and induction of pro-inflammatory IL-1β cytokine release^13^. Indeed, the ‘hemolysin’ activity of CyaA appears to counteract, to some extent, the immunosuppressive signaling of CyaA-produced cAMP. We have recently found that the pore-forming activity of CyaA plays a role in chemoattraction of neutrophils into the infected tissue and accounts for its inflammatory damage (Skopova *et al*. manuscript in preparation). The rather modest cell-permeabilizing activity of CyaA may thus reflect the evolution of an RTX hemolysin moiety for reduced harnessing of innate immune mechanisms and for serving primarily as a ‘molecular syringe’, delivering the immunosuppressively acting AC enzyme moiety into cytosol of myeloid phagocytes.

## Methods

### Construction, production and purification of CyaA proteins

Plasmid pT7CACT1 was used for co-expression of *cyaC* and *cyaA* genes allowing production of recombinant CyaC-activated CyaA in *Escherichia coli*^32^. Oligonucleotide-directed PCR mutagenesis was used to construct pT7CACT1-derived plasmids for expression of CyaA mutant variants harboring negatively or positively charged residues of the segment linking the AC and the hydrophobic domains substituted with asparagine, glutamine, lysine, serine, or alanine residues, respectively. Intact CyaA and its mutant variants were produced in *E. coli* XL1-Blue (Stratagene) transformed with appropriate pT7CACT1-derived constructs. Exponential 500-ml cultures were grown at 37 °C and induced by isopropyl 1-thio-β-D-galactopyranoside (IPTG, 1 mM) for 4 h before the cells were washed with 50 mM Tris-HCl (pH 8.0), 150 mM NaCl, resuspended in 50 mM Tris-HCl (pH 8.0), 0.2 mM CaCl_2_, and disrupted by sonication. Upon centrifugation at 25,000 × g for 20 min, the insoluble cell pellets were resuspended in 8 M urea, 50 mM Tris-HCl (pH 8.0), 50 mM NaCl, 0.2 mM CaCl_2_. Upon centrifugation at 25,000 × g for 20 min, clarified urea extracts were loaded onto a DEAE-Sepharose column equilibrated with 8 M urea, 50 mM Tris-HCl (pH 8.0), 120 mM NaCl. After washing, the CyaA proteins were eluted with 8 M urea, 50 mM Tris-HCl (pH 8.0), 2 M NaCl, diluted four times with 50 mM Tris-HCl (pH 8.0), 1 M NaCl buffer, and further purified on a phenyl-Sepharose column equilibrated with the same buffer. Unbound proteins were washed out with 50 mM Tris-HCl (pH 8.0), and the CyaA proteins were eluted with 8 M urea, 50 mM Tris-HCl (pH 8.0), 2 mM EDTA and stored at −20 °C. Concentrations of the purified CyaA proteins were determined by the Bradford assay (Bio-Rad, Hercules, USA) using bovine serum albumin as a standard.

### Construction, production and purification of d6His-GST-CyaA_411–490_ and CyaA_411–490_

A 278 bp nucleotide sequence encoding a segment comprising residues 411–490 of CyaA (CyaA_411–490_) was amplified by polymerase chain reaction (PCR) with the forward primer 5′-AAACCATG*GAAAACCTGTACTTCCAGGGC*TCGCGATCGTTCTCGTTGG-3′ containing a restriction site for NcoI (underlined) and a nucleotide sequence encoding a cleavage site (ENLYFQG, in italics) for tobacco-etch virus protease (TEV protease), and the reverse primer 5′-AAACTCGAGTTAGGATCCGGCCCGGCCGAATTG-3′ carrying a restriction site for XhoI (underlined). The purified PCR product was digested with NcoI and XhoI (New England Biolabs) and cloned into the NcoI-XhoI-digested expression vector pET42b (Merck). Next, the 45 bp XbaI-NdeI fragment of the pET42b-CyaA_411–490_ vector was replaced by the 188 bp XbaI-NdeI fragment encoding an N-terminal double His tag sequence in the pET42b-dHis d6His-GST-SPM construct^60^. The resulting plasmid allowed expression of the CyaA_411–490_ segment as an glutathione S-transferase (GST) fusion protein (d6His-GST-CyaA_411–490_) in *E. coli* BL-21 λ(DE3) cells. These were grown at 37 °C to OD_600_ = ~0.8, induced by IPTG (0.5 mM) and cultivated for additional 4 h. Then the cells were washed with 50 mM Tris-HCl (pH 8.0), 150 mM NaCl and disrupted by sonication. Non-broken cells were removed by centrifugation at 2,500 × g for 5 min and the supernatant was centrifuged at 25,000 × g for 20 min. The pellet was resuspended in 8 M urea, 50 mM Tris-HCl (pH 8.0) and the urea extract was clarified by centrifugation at 25,000 × g for 20 min. The supernatant was loaded onto Ni-Sepharose 6 Fast Flow resin (GE Healthcare) equilibrated with TU buffer (8 M urea, 50 mM Tris-HCl, pH 8.0). The column was washed with TU buffer supplemented with 50 mM imidazole and the d6His-GST-CyaA_411–490_ fusion was eluted with TU buffer containing 300 mM imidazole. The eluted fraction was diluted with 50 mM Tris-HCl (pH 8.0) to a final concentration of 3 M urea and mixed with TEV protease (1:20 w/w) for 16 h at 4 °C. The mixture was supplemented with solid urea to obtain final concentration of 8 M urea and the suspension was loaded onto a Ni-Sepharose column equilibrated with TU buffer. The CyaA_411–490_ segment was recovered in the flow-through fraction and concentrated on Amicon ultrafiltration disc (cut off 3 kDa).

### Cell binding, cell invasive and hemolytic activities on sheep erythrocytes

AC enzymatic activities were measured in the presence of 1 μM calmodulin as previously described^61^. One unit of AC activity corresponds to 1 μmol of cAMP formed per min at 30 °C, pH 8.0. Hemolytic activities were measured in TNC buffer (20 mM Tris-HCl at pH 7.4, 150 mM NaCl and 2 mM CaCl_2_) by determining the hemoglobin release in time upon CyaA incubations (10 μg/ml) with washed sheep erythrocytes (5 × 10^8^/ml in TNC), as previously described^9^. Erythrocyte binding and cell-invasive AC activities were determined as described in detail previously^9^,^62^. Briefly, sheep erythrocytes (5 × 10^8^ cells/ml) were incubated with CyaA (1 μg/ml) at 37 °C in TNC buffer. After 30 min, cell suspensions were washed three times in TNE buffer (20 mM Tris-HCl at pH 7.4, 150 mM NaCl and 5 mM EDTA) to remove unbound CyaA and divided in two aliquots. First aliquot was directly used to determine the amount of cell-associated AC activity (membrane-bound CyaA). The second aliquot was treated with 20 μg/ml of trypsin for 15 min at 37 °C in order to inactivate the extracellular AC toxin which did not translocate into cells. Soybean trypsin inhibitor (40 μg/ml) was added to the mixture to stop the reaction before the samples were washed three times with TNE buffer and used to determine the amount of cell-invasive AC activity. Activity of intact CyaA was taken as 100%.

### Binding and cAMP elevation of CyaA on J774A.1 cells

J774A.1 murine monocytes/macrophages (ATCC, number TIB-67) were cultured at 37 °C in a humidified air/CO_2_ (19:1) atmosphere in RPMI medium supplemented with 10% (v/v) heat-inactivated fetal bovine serum, penicillin (100 i.u./ml), streptomycin (100 μg/ml) and amphotericin B (250 ng/ml). Prior to assays, RPMI was replaced with D-MEM medium (1.9 mM Ca^2+^) without FCS and the cells were allowed to rest in D-MEM for 1 h at 37 °C in a humidified 5% CO_2_ atmosphere^44^. J774A.1 cells (10^6^) were incubated in D-MEM with 1 μg/ml of CyaA variants for 30 min at 4 °C, prior to removal of unbound toxin by three washes in D-MEM. After the transfer to a fresh tube, cells were lyzed with 0.1% Triton X-100 for determination of cell-bound AC enzyme activity. For intracellular cAMP assays, 2 × 10^5^ cells were incubated with CyaA for 30 min in D-MEM, the reaction was stopped by addition of 0.2% Tween-20 in 100 mM HCl, samples were boiled for 15 min at 100 °C, neutralized by addition of 150 mM unbuffered imidazole and cAMP was measured as previously described^50^.

### Determination of cytosolic potassium levels by inductively coupled plasma mass spectrometry (ICP–MS)

Cytosolic potassium concentration was analyzed as previously described^15^. Briefly, incubation of J774A.1 cells (10^6^/ml) with CyaA (500 ng/ml) was carried out for 5 and 10 min. The cells were washed with modified HBSS (140 mM NaCl, 2 mM CaCl_2_, 2 mM MgCl_2_, 10 mM Hepes-Na, 50 mM glucose, pH 7.4) and lysed with 500 ml of deionized water. The lysed cells were centrifuged (20 min, 4 °C, 40,000 × g) to remove the membranes and the soluble fraction was stored at −20 °C. ICP–MS measurements were carried out using an Agilent 7700x inductively coupled plasma mass spectrometer with ASX-500 autosampler, equipped with a Micro-Mist concentric nebulizer and High Matrix Interface. Potassium was detected at m/z 39, in a collision cell mode (He 4.8 ml/min), using 45Sc and 89Y as internal standards to correct for sensitivity drifts. Prior to the ICP–MS analysis, lysates were diluted approximately 10 times (weight) with deionized water. Quantification was performed using a five-point external calibration (0.1–2.0 mg/l K^+^, data not shown). Results were processed using Agilent Mass Hunter software.

### Flow cytometry

J774A.1 cells (2 × 10^5^/well) labeled with TMRE (40 nM) were incubated 2 h with CyaA, CyaA_D445N+D446N+E448Q_ or control buffer and stained with Hoechst 33258 (0.5 μg/ml). Live and necrotic cells were detected by flow cytometry on a FACS LSR II instrument (BD Biosciences, San Jose, CA) and data were analyzed using the FlowJo software (Tree Star, Ashland, OR).

### Identification of membrane-associated peptides using mass spectrometry (MS)

Multilamellar hand-shaken liposome vesicles (1 mg/ml) made from soybean phosphatidylcholine (SPC type IIS, phosphatidylcholine content 17%) and cholesterol (10:1 w/w) were prepared in 20 mM Hepes (pH 7.4), 150 mM NaCl and 2 mM CaCl_2_. Large unilamellar vesicles of mean size of 1000 nm (LUV 1000) were prepared by extrusion of hand-shaken liposome vesicles with the LiposoFast Basic apparatus (Avestin) equipped with a polycarbonate membrane of 1000 nm pore diameter (Avestin). The d6His-GST-CyaA_411–490_ fusion (200 μg/ml) was incubated at 37 °C with suspension of LUV 1000. After 30 min of incubation, the unbound d6His-GST-CyaA_411–490_ was removed by washing of liposomes with 20 mM Hepes (pH 7.4), 150 mM NaCl and 2 mM CaCl_2_, followed by centrifugation at 6,000 × g at 4 °C, using a fresh tube at each step to avoid d6His-GST-CyaA_411–490_ carry over on tube walls. Liposomes with bound d6His-GST-CyaA_411–490_ were treated with 1 μg/ml of sequencing-grade trypsin for 90 min at 37 °C. Soybean trypsin inhibitor at a concentration of 2 μg/ml (Promega, Madison, WI) was added to stop the reaction before the samples were washed once with 20 mM Hepes (pH 7.4), 150 mM NaCl and 2 mM EDTA, further with 0.1 M Na_2_CO_3_ (pH 10.5) and finally in ddH_2_O. For each washing step, the liposomes were resuspended in the indicated buffers and transferred to fresh polypropylene centrifugation tubes in order to avoid any carry-over of d6His-GST-CyaA_411–490_ fragment bound to tube walls instead of liposomes. The control in-solution digestion of d6His-GST-CyaA_411–490_ was done overnight at 37°C in a cleavage buffer containing 25 mM 4-ethylmorpholine acetate, 5% acetonitrile (MeCN) and sequencing-grade trypsin (100 ng). Prior to MS analysis, the peptides were extracted in 50 μl 0.2% trifluoroacetic acid (TFA), sonicated (20 min) and samples were further desalted using a GELoader (Eppendorf, Hamburg, Germany) microcolumn packed with a Poros Oligo R3 reversed-phase material (Applied Biosystems, Foster City, CA). The purified and concentrated peptides were eluted from the microcolumn in several droplets directly onto a MALDI plate using 1 μl of matrix solution (α-cyano-4-hydroxycinnamic acid (Bruker Daltonics) in 50% MeCN/0.1% TFA; 5 mg/ml). MALDI mass spectra were measured on a SolariX XR™ FT-ICR instrument equipped with a 12T superconducting magnet (Bruker Daltonics). The spectra were acquired in the mass range of 600–4000 Da and calibrated externally using a Peptide Calibration Standard II (Bruker Daltonics) resulting in a mass accuracy below 2 ppm.

### Lipid bilayer experiments

Measurements on planar lipid bilayers (black lipid membranes) were performed in Teflon cells separated by a diaphragm with a circular hole (diameter 0.5 mm) bearing the membrane. The CyaA toxin was diluted in 8 M urea, 50 mM Tris-HCl (pH 8.0), 2 mM EDTA and added into the grounded cis compartment with positive potential. The membrane was formed by the painting method using 3% soybean phosphatidylcholine (type IIS, asolectin; Sigma-Aldrich) in n-decane–butanol (9:1, vol/vol). Both compartments contained 150 mM KCl, 10 mM Tris, and 2 mM CaCl_2_ (pH 7.4), the temperature was 25 °C. The membrane current was registered by Ag/AgCl electrodes (Theta) with salt bridges (applied voltage, 50 mV), amplified by LCA-200-100 G amplifier (Femto), and digitized by use of a KPCI-3108 card (Keithly). The signal was analyzed using QuB software. The lifetimes of CyaA pores were calculated from ~1000 individual events from 5 to 10 different membranes for each mutant protein. We used two-exponential model to successfully fit the single-pore lifetime data (Equation (1)). The shorter lifetime (tau~100 ms) corresponds to fast fluctuation between conductive and closed pore states which is not crucial for overall membrane activity. We show only tau (>500 ms) in Table 2, that is responsible for majority of membrane conductance. The conductance data were processed with 10 Hz filter and the individual dwell times of opened state were found using QuB software. Kernel density estimates (KDE) of lifetimes were prepared using Gaussian kernel with a half width at half maximum (hwhm) of 75 ms using our own software. The KDE distribution was fitted with double exponential function in range of 75 ms to 10 s using Gnuplot.

For fitting we used the following function:


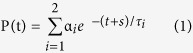


where t is the dwell time, α_1_ + α_2_ = 1 and s = 75 ms, and which represents the resolution of the fitted distribution.

### Circular dichroism (CD) spectroscopy

The far-UV CD spectra were recorded on a Jasco-815 spectropolarimeter in rectangular quartz Suprasil cells of 1-mm path length (110-QS, Hellma). The CyaA_411–490_ segment was rapidly diluted with 5 mM Tris-HCl (pH 7.4) or 5 mM Tris-HCl (pH 7.4), 1% n-dodecyl-β-D-maltoside (DM), and 5 mM Tris-HCl (pH 7.4), 50% trifluoroethanol (TFE) to obtain a final protein concentration of 100 μg/ml. The samples were measured for wavelengths from 200 to 280 nm at 20 °C at standard instrument sensitivity and scanning speed of 10 nm/min, response time of 16 ms and two spectra accumulations. The resulting spectra were obtained by subtracting the spectrum of the buffer from the spectrum of the protein solution. Secondary structure composition of CyaA_411–490_ was calculated by method of Micsonai *et al*.^63^.

### Statistical analysis

Significance of differences in values was assessed by Student’s t-test.

## Additional Information

**How to cite this article**: Masin, J. *et al*. Negatively charged residues of the segment linking the enzyme and cytolysin moieties restrict the membrane-permeabilizing capacity of adenylate cyclase toxin. *Sci. Rep.* **6**, 29137; doi: 10.1038/srep29137 (2016).

## Supplementary Material

Supplementary Information

## Figures and Tables

**Table 1:** MALDI FT-ICR MS identification of peptide fragments of the d6His-GST-CyaA_411–490_ protein associated with the liposome membrane.

AA position	Peptide sequence	MH^+^ theor.	MH^+^ exp.	Error [ppm]
138–151#	AEISMLEGAVLDIR	1516.8040	1516.8024	1.1
138–158#	AEISMoxLEGAVLDIRYGVSR	2095.0852	2095.0852	0
306–324#	QHMDSPDLGTGGGSGIEGR	1870.8348	1870.8349	0.1
325–333#, 411–413+	GSMENLYFQGSR	1388.6263	1388.6260	0.2
414–435+	SFSLGEVSDMAAVEAAELEMTR	2343.0843	2343.0825	0.8
436–443+	QVLHAGAR	851.4846	851.4842	0.5
444–461+	QDDAEPGVSGASAHWGQR	1867.8318	1867.8309	0.5
462–474+	ALQGAQAVAAAQR	1254.6913	1254.6911	0.2
475–487+	LVHAIALMTQFGR	1456.8093	1456.8083	0.7
462–490+	ALQGAQAVAAAQRLVHAIALMTQFGRAGS	2907.5734	2907.5714	0.7

^#^Observed peptides corresponding to the glutathione S-transferase (GST). The amino acid numbering is derived from the original d6His-GST sequence (1–333).

^+^Identified peptide fragments corresponding to the segment 411 to 490 of CyaA. The amino acid numbering of the peptides derived from the linker sequence (411–490) is according to the sequence of full-length CyaA.

**Table 2:** Activities of the CyaA mutant variants on lipid bilayers.

Toxin	Pore lifetime τ (ms)^a^	Overall membrane activity^b^
CyaA	1198	+
CyaA_E419Q−E432Q_	2399	+++
CyaA_E419K−E432K_	1376	+++++
CyaA_D445N+D446N+E448Q_	1498	+++
CyaA_D445K+D446K+E448K_	1228	++
CyaA_D445N_	505	+
CyaA_D446N_	1804	++
CyaA_E448Q_	1246	++

^a^The lifetimes of open pore states τ of CyaA were estimated from ~1000 individual events for each mutant protein as specified in Methods as equation (1).

^b^The overall membrane activity was detected after 5 min incubation of the membranes with individual proteins at 1 nM concentration. The number of plus signs refers to the overall conductance of the membrane/cm^2^ induced by the various CyaA proteins under these conditions in asolectin membranes and reflects the size (conductance), the lifetime, and the specific frequency of formation of pores by the various CyaA constructs.
