# Supplementary material for: Negatively charged residues of the segment linking the enzyme and cytolysin moieties restrict the membrane-permeabilizing capacity of adenylate cyclase toxin
Source: Sci Rep. 2016 Sep 1;6:29137. doi: 10.1038/srep29137 (PMC5007505; doi:10.1038/srep29137)
Supplement: Supplementary Information [file srep29137-s1.pdf]

Supplementary Information

Negatively charged residues of the segment linking the enzyme and cytolysin moieties restrict the membrane-permeabilizing capacity of adenylate cyclase toxin

Jiri Masin, Adriana Osickova, Anna Sukova, Radovan Fiser, Petr Halada, Ladislav Bumba, Irena Linhartova, Radim Osicka and Peter Sebo

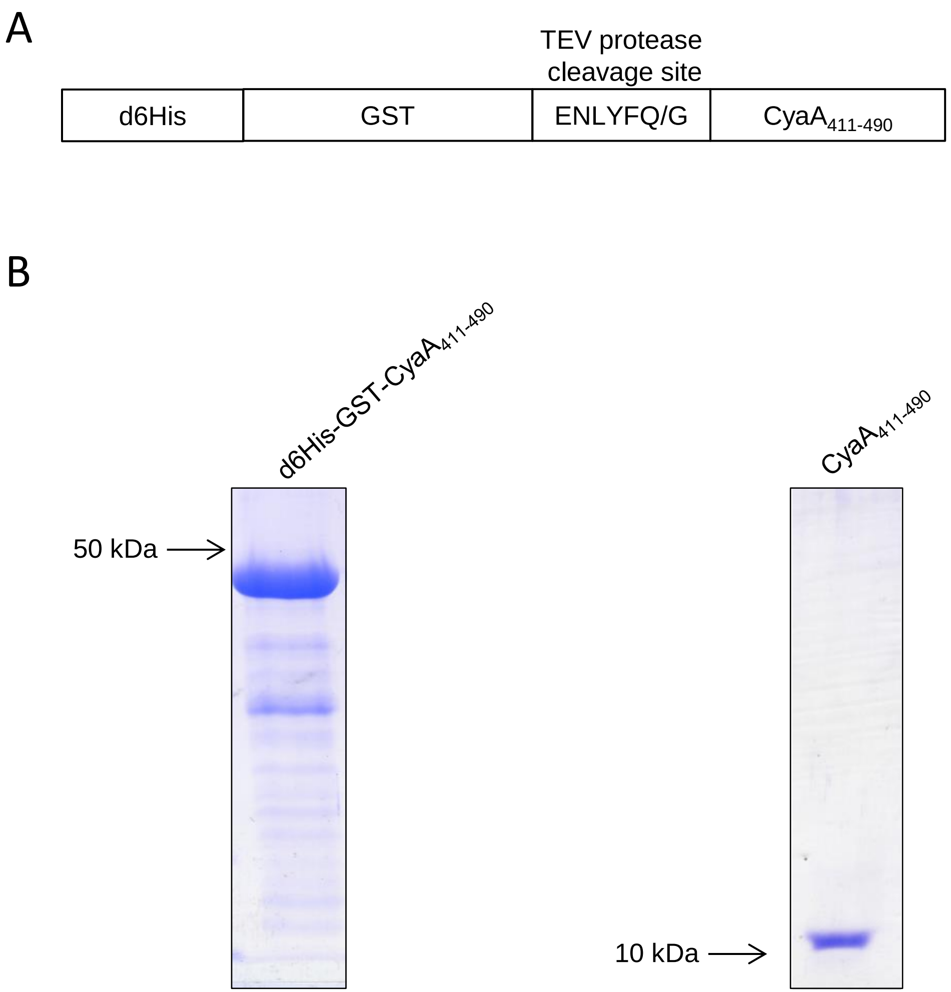

Supplementary Figure S1. A) Schematic representation of the d6His-GST-CyaA$_{411\text{-}490}$ fusion protein. B) The d6His-GST-CyaA$_{411\text{-}490}$ was recovered from inclusion bodies by 8 M urea treatment after induction of *E. coli* cells with 0.5 mM IPTG. The urea extract was loaded on a Ni-NTA agarose column and d6His-GST-CyaA$_{411\text{-}490}$ was eluted with 600 mM imidazole in 8 M urea (left panel). The eluted fraction was diluted to a final concentration of 2 M urea and incubated with TEV protease (1:20 w/w) for 16 h at 4°C. The mixture was concentrated on Amicon ultrafiltration disc (cut off 10 kDa) and the soluble CyaA$_{411\text{-}490}$ was recovered in the permeate. The retentate was mixed with solid urea to obtain final concentration of 8 M urea and the suspension was loaded on a Ni-NTA agarose column. The CyaA$_{411\text{-}490}$ was obtained as the flow-through fraction from the Ni-NTA agarose column (right panel).

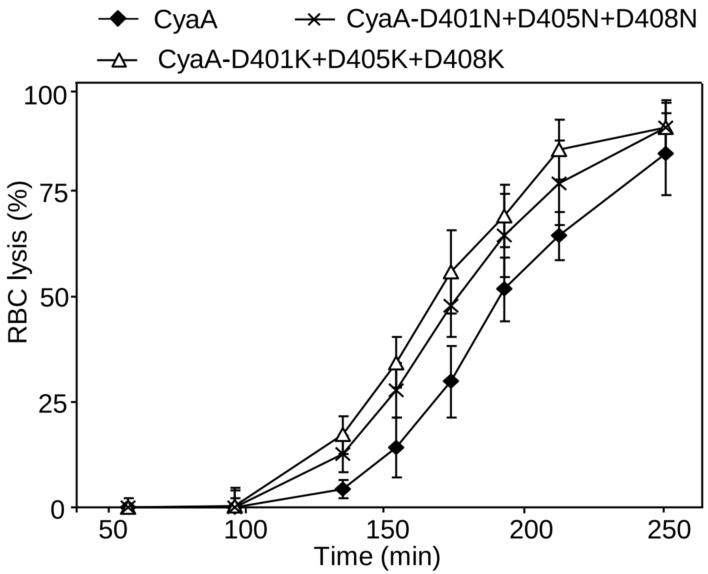

Supplementary Figure S2. **Substitutions of glutamate and aspartate residues in block I of the 'AC to Hly-linking segment' moderately enhance pore forming capacity of the CyaA mutants**. Sheep erythrocytes (5x10^8/ml) resuspended in TNC buffer were incubated at 37°C in the presence of intact CyaA or its mutant variants (10 µg/ml). Hemolytic activity was measured as the amount of released hemoglobin by photometric determination (A_{541nm}). Error bars represent standard deviations from three independent measurements.

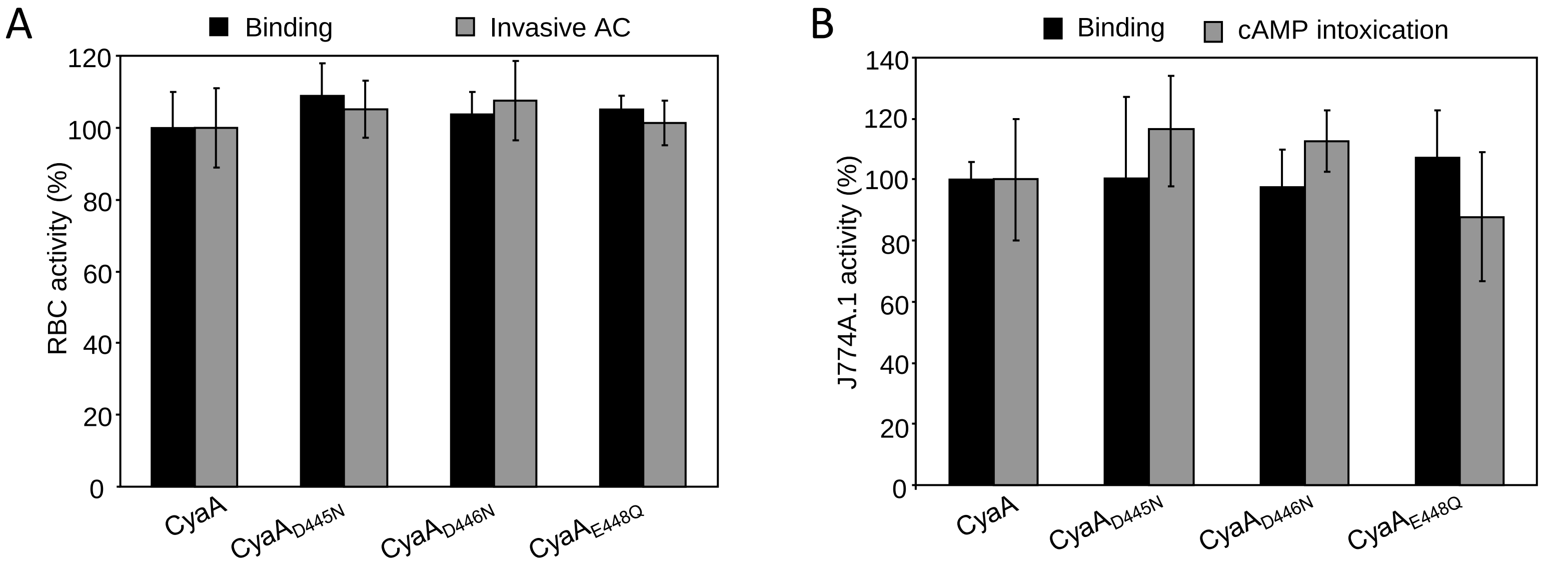

Supplementary Figure S3. Single substitutions of aspartate and glutamate residues in block III of the 'AC to Hly-linking' segment have no effect on membrane binding capacity or AC domain translocation of the CyaA mutants. A) Sheep erythrocytes ($5x10^8$/ml) were incubated at 37°C with 1 µg/ml of the purified CyaA proteins and after 30 min, aliquots were taken for determinations of the cell-associated AC activity and of the AC activity internalized into erythrocytes and protected against digestion by externally added trypsin. Activities are expressed as percentages of intact CyaA activity and represent average values ± standard deviations from two independent determinations performed in duplicate (n=4). B) CyaA binding to J774A.1 cells (10^6) was determined as the amount of total cell-associated AC enzyme activity upon cells incubation with 1 µg/ml of CyaA for 30 min at 4°C. cAMP intoxication was assessed by determining the intracellular concentration of cAMP generated in cells after incubation of J774A.1 cells ($2x10^5$) with four different toxin concentrations from within the linear range of the dose-response curve (100, 50, 25, 10 ng/ml). The percentage of cAMP accumulation in cells at each toxin concentration was calculated, taking cAMP values for intact CyaA as 100%. All activities are expressed as percentages of intact CyaA activity and represent average values ± standard deviations from two independent determinations performed in duplicate (n=4).

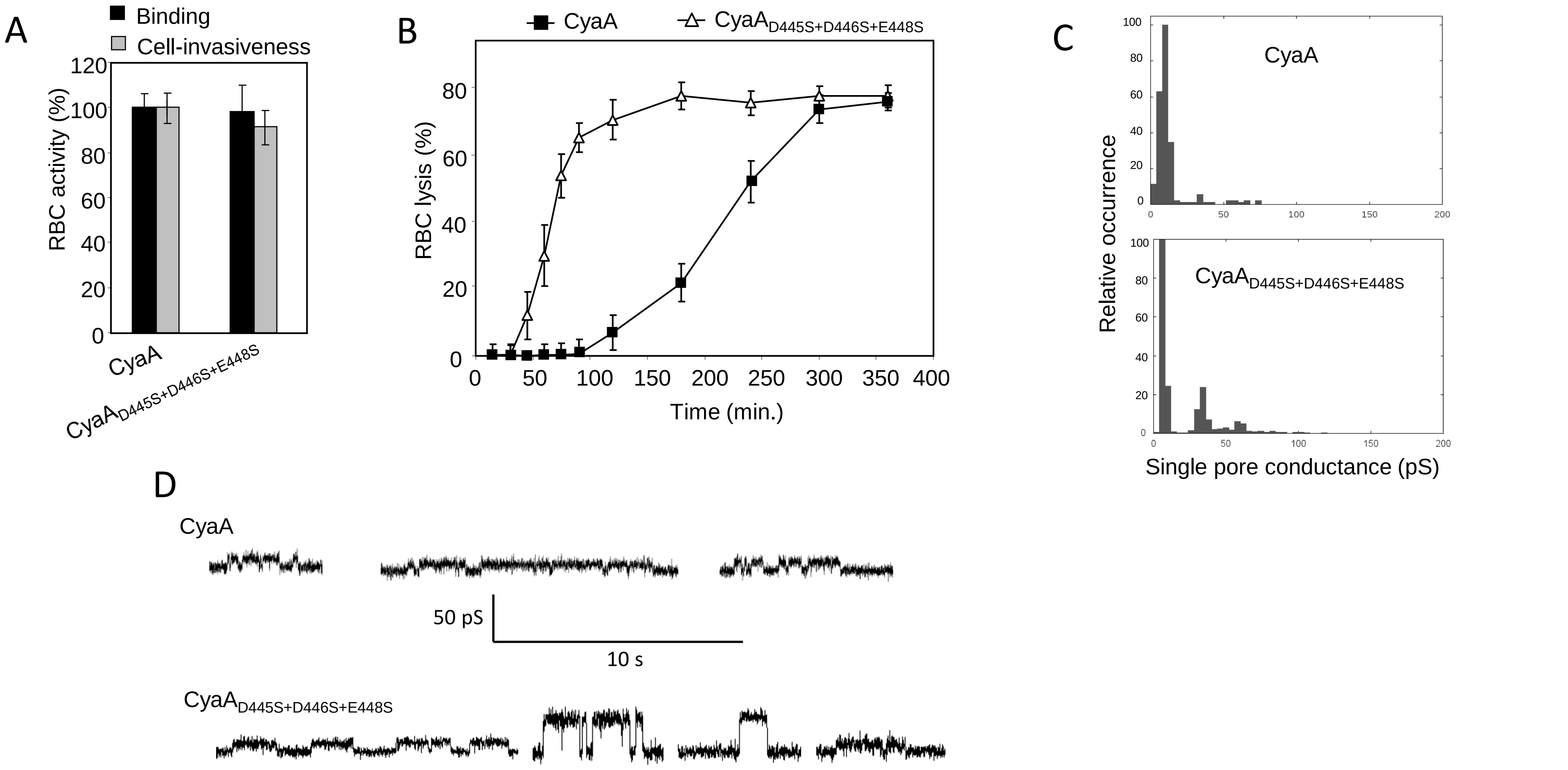

Supplementary Figure S4. Substitutions of glutamate and aspartate residues by three serine residues in block III enhance pore-forming capacity of CyaA without altering AC translocation capacity. A) Sheep erythrocytes (5 x 10^8/ml) were incubated at 37°C with 1 µg/ml of the purified CyaA proteins and after 30 min, aliquots were taken for determinations of the cell-associated AC activity and of the AC activity internalized into erythrocytes and protected against digestion by externally added trypsin. Activities are expressed as percentages of intact CyaA activity and represent average values ± standard deviations from two independent determinations performed in duplicate with two different toxin preparations. B) Sheep erythrocytes (5x10^8/ml) in TNC buffer were incubated at 37°C in the presence of intact CyaA or $CyaA_{D445S+D446S+E448S}$ (10 µg/ml). Hemolytic activity was measured as the amount of released hemoglobin by photometric determination (A541nm). Error bars represent standard deviations from three independent measurements. C) Single-pore conductance of intact CyaA and $CyaA_{D445S+D446S+E448S}$ (1 nM) was determined in 150 mM KCl, 10 mM Tris-HCl and 2 mM $CaCl_2$ (pH 7.4) at 25°C and membrane potential -50 mV. D) Single pore recordings for CyaA and mutated forms. The representative recordings of most frequent types of observed pores. The presented events originate from several different asolectin membranes for each toxin. Measurement conditions: 150 mM KCl, 10 mM Tris, 2 mM $CaCl_2$, pH 7.4, toxin concentration 0.1-1 nM, transmembrane potential -50mV.

Supplementary Table 1: MALDI FT-ICR MS peptide mass mapping data of in-solution tryptic digest of d6His-GST-CyaA$_{411-490}$ fusion protein.

AA position	Peptide sequence	MH^+ theor.	MH^+ exp.	Error [ppm]
1-11#	MVPHHHHHHSR	1411.6661	1411.6667	0.4
47-57#	LHMSPILGYWK	1344.7133	1344.7140	0.5
67-83#	LLLEYLEEKYEEHLYER	2269.1387	2269.1378	0.4
67-90#	LLLEYLEEKYEEHLYERDEGDKWR	3155.5320	3155.5285	1.1
84-90#	DEGDKWR	905.4112	905.4121	1.0
113-121#	LTQSMAIIR	1032.5870	1032.5880	1.0
136-151#	ERAEISMLEGAVLDIR	1801.9477	1801.9504	1.5
138-151#	AEISMLEGAVLDIR	1516.8040	1516.8059	1.3
180-188#	MFEDRLCHK	1178.5445	1178.5454	0.8
230-239#	RIEAIPQIDK	1182.6841	1182.6858	1.4
246-266#	YIAWPLQGWQATFGGGDHPPK	2326.1404	2326.1432	1.2
325-333#, 411-413+	GSMENLYFQGSR	1388.6263	1388.6272	0.6
414-435+	SFSLGEVSDMAAVEAAELEMTR	2343.0843	2343.0805	0.3
436-443+	QVLHAGAR	851.4846	851.4848	0.2
444-461+	QDDAEPGVSGASAHWGQR	1867.8318	1867.8341	1.2
462-474+	ALQGAQAVAAAQR	1254.6913	1254.6924	0.9
475-487+	LVHAIALMTQFGR	1456.8093	1456.8108	1.0

#Observed peptides corresponding to the glutathione S-transferase (GST). The amino acid numbering is derived from the original GST sequence (1-333).

+Identified peptide fragments corresponding to the segment 411 to 490 of CyaA. The amino acid numbering of the peptides derived from the linker sequence (411-490) is according to the sequence of full-length CyaA.

Supplementary Table 2: Secondary structure composition calculated from CD spectra of $CyaA_{411\text{-}490}$.

	Helix (%)	Sheet (%)	Turn (%)	Other (%)
**Buffer**	6.6	20.8	17.5	55.1
**Dodecyl maltoside**	26.7	14.6	16.3	42.3
**Trifluoroethanol**	51.9	8.2	12.0	28.0

Secondary structure composition of $CyaA_{411\text{-}490}$ was calculated by BeStSel algorithm (Micsonai *et al.*, 2015).
